# Circulating levels of PCSK9, ANGPTL3 and Lp(a) in stage III breast cancers

**DOI:** 10.1186/s12885-022-10120-6

**Published:** 2022-10-06

**Authors:** Emilie Wong Chong, France-Hélène Joncas, Nabil G. Seidah, Frédéric Calon, Caroline Diorio, Anne Gangloff

**Affiliations:** 1 Faculty of Medicine, Laval University, Quebec City, QC Canada; 2 Oncology Research Axis, CHU de Québec-Laval University Research Center, Quebec City, QC Canada; 3 Cancer Research Centre (CRC), Laval University, Quebec City, QC Canada; 4 Laboratory of Biochemical Neuroendocrinology, Institut de Recherches Cliniques de Montréal, Montreal, QC Canada; 5 Faculty of Pharmacy, Laval University, Quebec City, QC Canada; 6 Neuroscience Research Axis, CHU de Québec-Laval University Research Center, Quebec City, QC Canada; 7 Centre Des Maladies du Sein, Hôpital du Saint-Sacrement, Quebec City, QC Canada; 8 Lipid Clinic, CHU de Québec, Quebec City, QC Canada

**Keywords:** ANGPTL3, Lipoprotein(a), PCSK9, Breast cancer, Endogenous lipid metabolism, Cancer progression

## Abstract

**Background / synopsis:**

Cholesterol and lipids play an important role in sustaining tumor growth and metastasis in a large variety of cancers. ANGPTL3 and PCSK9 modify circulating cholesterol levels, thus availability of lipids to peripheral cells. Little is known on the role, if any, of circulating lipid-related factors such as PCSK9, ANGPTL3 and lipoprotein (a) in cancers.

**Objective/purpose:**

To compare circulating levels of PCSK9, ANGPTL3, and Lp(a) in women with stage III breast cancer *versus* women with premalignant or benign breast lesions.

**Methods:**

Twenty-three plasma samples from women diagnosed with a stage III breast cancer (ductal, lobular or mixed) were matched for age with twenty-three plasma samples from women bearing premalignant (stage 0, *n* = 9) or benign (*n* = 14) breast lesions. The lipid profile (Apo B, total cholesterol, HDL cholesterol and triglycerides levels) and Lp(a) were measured on a Roche Modular analytical platform, whereas LDL levels were calculated with the Friedewald formula. ANGPTL3 and PCSK9 plasma levels were quantitated by ELISA. All statistical analyses were performed using SAS software version 9.4.

**Results:**

PCSK9 levels were significantly higher in women with stage III breast cancer compared to age-matched counterparts presenting a benign lesion (95.9 ± 27.1 ng/mL *vs*. 78.5 ± 19.3 ng/mL, *p* < 0.05, *n* = 14). Moreover, PCSK9 levels positively correlated with breast disease severity (benign, stage 0, stage III) (Rho = 0.34, *p* < 0.05, *n* = 46). In contrast, ANGPTL3 and Lp(a) plasma levels did not display any association with breast disease status and lipids did not correlate with disease severity.

**Conclusion:**

In this small cohort of 46 women, PCSK9 levels tended to increase with the severity of the breast disease. Given that PCSK9 plays an important role in maintaining cholesterolemia, and a potential role in tumor evasion, present results warrant further investigation into a possible association between PCSK9 levels and breast cancer severity in larger cohorts of women.

## Introduction

Growing evidence suggests a relationship between cholesterol availability and cancer progression [1–4]. Breast cancer is no exception, with preclinical data supporting the important role of cholesterol to sustain cancer development and progression [5–9]. Circulating levels of lipids do not adequately reflect the presence of cancer, with discrepant data published on the association between circulating levels of cholesterol and breast cancer. Some studies described an association between low-density lipoprotein cholesterol (LDL-C) and breast cancers [8, 10, 11], while others showed no association [12, 13] or an inverse relationship [14–16]. Circulating cholesterol levels are a function of several concomitant processes such as synthesis, absorption, distribution, uptake and degradation. Circulating factors such as PCSK9 [17–20] and ANGPTL3 [21, 22] also contribute to the regulation of cholesterol levels. The lack of robust association between cholesterolemia and breast cancer might be explained by the interplay between the above-mentioned processes. Since the role, if any, of PCSK9, ANGPTL3, and Lp(a) in cancers has not been fully investigated to date, the current study aims to establish the levels of these lipid-related proteins in breast cancers.

PCSK9 triggers hepatic LDL-receptor (LDLR) degradation [23] resulting in higher circulating levels of LDL-C [24, 25]. Inhibition of PCSK9 protects LDLR, the latter are recycled at the surface of the hepatocytes each time they internalize LDL cholesterol, resulting in lower circulating levels of LDL-C. Although PCSK9 and its inhibition have been principally studied in atherosclerosis and cardiovascular disease [17–20], a growing number of reports suggest a role of PCSK9 in cancers [26–30], not only as a cholesterol supplier, but also as an immune response down-regulator, by triggering degradation of the Major Histocompatibility Complex 1 (MHC-I) receptor [31–33]. Inhibition of PCSK9 not only protects LDLR but also MHC-I from PCSK9-induced degradation [34]. It has been shown in animal models of breast cancers that increased levels of MHC-I enhance both natural immunity and immunotherapies against cancer [35, 36]. Cholesterol metabolism disruption, by combining PCSK9 inhibition with ezetimibe and a statin, is currently under investigation in patients with metastatic pancreatic cancers (NCT 04862260), while PCSK9 inhibition and its effect on MHC-I is being studied in patients with glioblastomas (NCT 04937413).

Angiopoietin-like protein 3 (ANGPTL3) is an inhibitor of both lipoprotein lipase and hepatic lipase [37–40]. Individuals with ANGPTL3 loss-of-function have higher lipoprotein lipase activity and display low blood levels of triglycerides, LDL and HDL-cholesterol, a condition known as familial combined hypocholesterolemia [21, 22]. This condition prompted the development of ANGPTL3 inhibitors to treat an almost opposite phenotype: homozygous familial hypercholesterolemia [41–43]. Little has been documented on the link between ANGPTL3 and cancers, yet some associations have been reported [44, 45].

Lp(a) is an LDL-like molecule that has caught a lot of attention these past few years in the field of cardiometabolic diseases [46–48]. Elevated levels of Lp(a) represent an emerging risk factor for atherosclerosis and thrombogenic events [49–51]. Lp(a) levels are genetically determined [52] and do not correlate with LDL-cholesterol levels. Association between certain types of cancers, including hormone-dependent breast cancers, and Lp(a) levels are inconsistent, with some studies describing a link [53–57], while others report no association with cancers [58].

Monoclonal antibodies and/or antisense therapy directed against PCSK9 and ANGPTL3 are FDA approved in other indications than cancers (cardiovascular disease, familial hypercholesterolemia), while inhibitors of Lp(a) are currently investigated in clinical trials (ex: Phase 3 trial NCT04023552). If future studies reveal that PCSK9, ANGPTL3 or Lp(a) have a role to play in cancer progression, the availability of inhibitors -for which safety data are available in humans- could permit to reposition these inhibitors in cancers. To date, whether these lipid-related factors have any role to play in cancers is still under investigation.

The present work aimed to determine plasma levels of PCSK9, ANGPTL3 and Lp(a) in women with benign disease of the breast, stage 0 and stage III breast cancers.

## Material and method

### Subjects and blood samples

Our study was performed using plasma samples from the registered biobank of the *Centre des maladies du sein Deschênes-Fabia* (Quebec City, Canada), collected from women aged 36 years or older, diagnosed with a benign breast disease (*n* = 14), stage 0 (*n* = 9) or stage III breast tumor (*n* = 23). Stage 0 breast tumors are non-invasive and found only in the lining of the lactiferous ducts, i.e., without spreading into the surrounding breast tissue. Stage III breast cancers are non-metastatic tumors that invade nearby tissues (ex: lymph nodes, muscles). Plasmas from patients with stage III breast cancers (cases) were age-matched with plasmas from women with benign lesion or stage 0 breast cancer (controls). Cancer stage was evaluated using the TNM system [59, 60]. Stage III breast tumors were either ductal, lobular or mixed. Body mass index (BMI) was extracted from medical records. Plasmas (EDTA) were collected between 2010 and 2014 from non-fasting patients and prior to initiation of cancer therapy. Samples were centrifuged to allow plasma separation, aliquoted and stored at -80 °C. Samples were frozen and thawed only once, the day of the experiments. Informed consent was obtained from all participants and ethics approval was obtained from the ethics committee of the CHU de Québec (2021–5624).

### Assessment of the lipid profile, Apo B and Lp(a)

Samples were sent to the Core laboratory of the *Hôpital de l’Enfant-Jésus* (Quebec City, Canada) for measurements of apolipoprotein B100 (Apo B, g/L), Lipoprotein (a) abbreviated as Lp(a) (nmol/L), total cholesterol (mmol/L), HDL-cholesterol (mmol/L) and triglycerides (mmol/L) using the Modular analytical platform (Roche). LDL-cholesterol was calculated with the Friedewald formula: LDL-cholesterol = total cholesterol – (triglycerides/ 2.2) – HDL in mmol/L. Non-HDL cholesterol was calculated by subtraction of HDL-cholesterol from total cholesterol in mmol/L. Lp(a) measurements lower than the reportable range (Lp(a) < 10 nmol/L) were set to a default value of 3 nmol/L (1/2 of the lower limit of detection) for statistical analyses. Insulin and C-peptide (pmol/L) were measured at the *Centre Hospitalier de l’Université Laval* (Quebec City, Canada) on a Centaur instrument (Siemens).

### ELISA for ANGPTL3 and PCSK9

ELISA kits to measure ANGPTL3 in ng/mL (Abcam, Cambridge, MA, USA) and PCSK9 levels in ng/mL (Biolegend, San Diego, CA, USA) were used according to manufacturers’ instructions with all supplied reagents. For ANGPTL3 quantitation, 50 µL of reconstituted ANGPTL3 standards or 1:120 diluted samples were added in duplicates into the 96-well ELISA plate. Fifty µL of antibody cocktail, prepared from a dilution of anti-human ANGPTL3 capture antibodies with anti-human ANGPTL3 HRP-coupled detector antibodies, were added to all wells, following which the plate was incubated 1 h at room temperature on a plate shaker. No blocking step was required. After incubation, the plate was washed 3 times. HRP substrate (TMB development solution) was added to wells and allowed to incubate in the dark for 10 min. To avoid HRP enzymatic signal saturation, the stop solution was added before reaching an optical density at 600 nm (OD_600_) of 1. OD_450_ was recorded as endpoint reading. For PCSK9 quantitation, samples were diluted 1:60. A standard curve was established with serial dilutions of reconstituted human PCSK9 in the assay diluent solution provided by the manufacturer. Anti-human PCSK9 pre-coated 96-well microplate was washed 4 times with washing buffer. Fifty µL of assay buffer was added per well prior to loading 50 µL of standard or diluted samples in duplicates on the plate. No blocking step was required. The plate was incubated at room temperature for 2 h, then washed 4 times before addition of 100 µL of anti-human PCSK9 detection antibody. A 1-h incubation followed. After 4 more washes, avidin-HRP solution was added to each well and allowed to incubate for 30 min. The plate was washed 6 times preceding addition of the substrate solution followed by a 15-min incubation in the dark. The HRP chromogenic reaction was quenched with a stop solution and OD was recorded at 450 nm.

### Statistical analysis

Data are presented in tables as means with standard deviations and as medians with interquartile ranges. Data analysis was performed by comparing cases to age-matched controls using the statistical softwares R version 4.0.4 and SAS version 9.4. Differences in the mean values between the case and control groups were analyzed using paired Student T tests, if normally distributed. If not, differences between measurements (cases vs controls) were compared using the Wilcoxon signed rank tests.

The control group was composed of benign disease of the breast and stage 0 breast cancers. One-way analyses of variances (ANOVA) or their non-parametric equivalents, Kruskal–Wallis H tests, were applied to assess differences between benign, stage 0 and stage III groups.

To assess whether the severity of the breast disease (benign breast disease, stage 0 breast cancer, stage III breast cancer) was associated with the variables under study (PCSK9, ANGPTL3, lipid profile components), we performed partial Spearman correlation tests adjusted for age and BMI. Results are displayed as graphical diagrams generated with the R software version 4.0.4 for Windows.

## Results

### Biochemical parameters of the control and the stage III breast cancer (cases) groups

Table 1 shows the characteristics of patients with a benign disease of the breast (*n* = 14) or a stage 0 breast cancer (*n* = 9), which together formed the control group (*n* = 23). No statistically significant differences were obtained for age, BMI, total cholesterol, triglycerides, LDL-C, non-HDL cholesterol, Lp(a), Apo B, PCSK9, ANGPTL3, non-fasting C-peptide or insulin. However, a difference in HDL-C was observed between benign disease (mean 1.3 mmol/L; SD ± 0.4 mmol/L, *n* = 14) and stage 0 breast cancer (mean 1.8 mmol/L; SD ± 0.4 mmol/L, *n* = 9), with overlapping values when considering standard deviations. Characteristics of the stage III breast cancer group (*n* = 23) *versus* the control group (*n* = 23) are presented in Table 2 as mean and median values. No significant differences were observed between case and control groups for age, BMI, lipid profile, non-fasting C-peptide and non-fasting insulin (*p*-values > 0.05 for the two-group comparison).

**Table 1:** Biological and biochemical characteristics of the control group stratified by breast disease type: benign breast disease and stage 0 breast tumor

Variable	Benign (*n* = 14)		Stage 0 (*n* = 9)		Two-group comparison
	Mean ± SD	Median ± IQR	Mean ± SD	Median ± IQR	*P*-value
**Age (years)**	54	54	58	51	0.87‡
	± 9	[50; 62]	± 6	[50; 60]	
**BMI (kg/m**^2^**)**	26.0	26.0	24.0	23.9	0.28†
	± 4.9	[21.1; 30.8]	± 3.0	[22.7; 24.5]	
**Cholesterol (mmol/L)**	5.0	4.8	5.2	5.3	0.59†
	± 1.0	[4.4; 5.9]	± 0.4	[5.0; 5.3]	
**Triglycerides (mmol/L)**	1.8	1.5	1.1	1.0	0.15‡
	± 1.1	[1.0; 2.0]	± 0.3	[0.8; 1.4]	
**HDL-C (mmol/L)**	1.3	1.3	1.8	1.8	0.01†*
	± 0.4	[1.1; 1. 6]	± 0.4	[1.4; 2.2]	
**LDL-C (mmol/L)**	2.9	2.8	2.9	3.1	0.95†
	± 1.2	[2.2; 3.8]	± 0.7	[2.3; 3.3]	
**Non-HDL (mmol/L)**	3.7	3.5	3.4	3.6	0.52†
	± 1.1	[3.0; 4.6]	± 0.8	[2.8; 3.8]	
**Lp(a) (nmol/L)**	55	33	86	47	0.36‡
	± 58	[11; 89]	± 92	[17; 161]	
**Apo B (g/L)**	1.00	0.97	0.95	1.00	0.71†
	± 0.32	[0.78; 1.26]	± 0.2	[0.76; 1.09]	
**PCSK9 (ng/mL)**	78.5	76.7	100.1	85.4	0.09†
	± 19.3	[65.2; 94.0]	± 39.0	[68.7; 142.3]	
**ANGPTL3 (ng/mL)**	94.0	93.1	104.1	110.4	0.50†
	± 36.2	[59.4; 132.9]	± 31.3	[78.2; 128.0]	
**Non-fasting C-peptide (pmol/L)**	860	882	726	554	0.51†
	± 495	[356; 1093]	± 432	[371; 982]	
**Non-fasting Insulin (pmol/L)**	122	124	97	44	0.60‡
	± 88	[25; 186]	± 95	[32; 140]	

*SD* Standard Deviation, *IQR* Interquartile range

^†^Unpaired Student T-test

^‡^Unpaired Wilcoxon Mann Whitney U test

^*^p-value < 0.05

**Table 2:** Characteristics of the whole cohort: stage III breast tumor *vs* control participants (benign disease of the breast pooled with stage 0 breast cancer)

Variable	Control (*n* = 23) Benign and Stage 0		Case (*n* = 23) Stage III		Case–Control comparison *p*-values	
	Mean ± SD	Median ± IQR	Mean ± SD	Median ± IQR	Unpaired	Paired
**Age (years)**	54	54	55	54	0.86†	0.12^₮^
	± 7.7	[50; 62]	± 7.3	[51; 62]		
**BMI (kg/m**^2^**)**	25.2	24.7	26.2	25.7	0.46†	0.45^₮^
	± 4.3	[21.4; 30.0]	± 4.6	[23.1; 28.4]		
**Triglycerides (mmol/L)**	1.5	1.3	1.5	1.5	0.33‡	0.77^₩^
	± 1.0	[0.8; 1.7]	± 0.5	[1.2; 1.5]		
**Cholesterol (mmol/L)**	5.1	5.1	5.0	5.1	0.77‡	0.79^₩^
	± 0.8	[4.7; 5.4]	± 1.0	[4.1; 5.6]		
**HDL-C (mmol/L)**	1.5	1.5	1.5	1.4	0.77†	0.75^₮^
	± 0.4	[1.2; 1.8]	± 0.4	[1.2; 1.7]		
**LDL-C (mmol/L)**	2.9	3.0	2.9	2.7	0.90†	0.92^₮^
	± 1.0	[2.3; 3.4]	± 0.8	[2.1; 3.6]		
**Non-HDL (mmol/L)**	3.6	3.5	3.6	3.6	0.87†	0.89^₮^
	± 0.9	[3.0; 4.0]	± 0.9	[2.6; 4.3]		
**Apo B (g/L)**	1.0	1.0	1.0	1.0	0.87†	0.88^₮^
	± 0.3	[0.8; 1.1]	± 0.2	[0.8; 1.1]		
**Lp(a) (nmol/L)**	67	42	94	35	0.97‡	0.51^₩^
	± 72.9	[13; 136]	± 117.1	[12; 169]		
**PCSK9 (ng/mL)**	87.0	81.7	98.9	97.9	0.056‡	0.065^₩^
	± 29.8	[67.5; 104.2]	± 23.5	[84.1; 117.5]		
**ANGPTL3 (ng/mL)**	98.0	95.0	108.2	104.1	0.36†	0.33^₮^
	± 34.0	[70.0; 131.3]	± 41.5	[85.3; 130.9]		
**Non-fasting C-peptide (pmol/L)**	808	856	799	652	0.81‡	0.99^₩^
	± 466	[398; 1066]	± 500.3	[437; 1088]		
**Non-fasting Insulin (pmol/L)**	112	106	121	72	0.95‡	0.75^₩^
	± 89.7	[31; 160]	± 114	[37; 218]		

*SD* Standard Deviation, *IQR* Interquartile range

^†^Unpaired Student T-test

^‡^Unpaired Wilcoxon Mann Whitney U test

^₮^Paired Student T-test

^₩^Paired Wilcoxon signed rank test

### Circulating levels of PCSK9, ANGPTL3 and Lipoprotein (a) in Stage III breast cancer *vs* control group

**Fig. 1:**
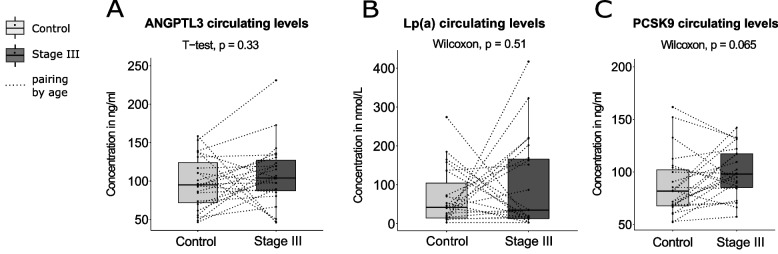
ANGPTL3, Lp(a) and PCSK9 distributions between control and stage III breast cancer groups. Boxplots represent the plasma levels of each circulating factor with median and interquartile range. Dotted lines connect age-matched individuals. Student T-test and Wilcoxon signed rank test did not yield any significant difference between stage III breast cancer and control plasma level distributions (*p* > 0.05). Stage III constituted the case group (*n* = 23). Control group combined benign pathology of the breast (*n* = 14) with stage 0 breast cancers (*n* = 9)

**Fig. 2:**
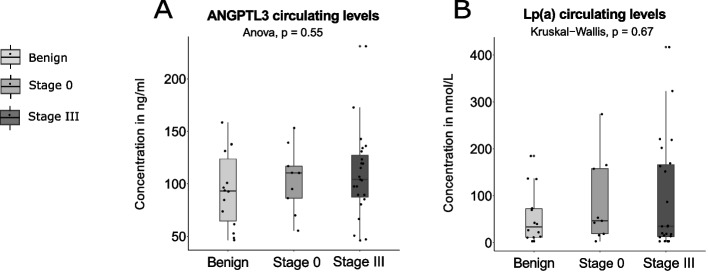
**A** ANGPTL3 and (**B**) Lp(a) circulating level distributions after control group stratification. Distributions between benign disease of the breast (*n* = 14), stage 0 (*n* = 9) and stage III (*n* = 23) breast tumor groups are represented as box plots. No significant difference was found following group comparison with ANOVA or Kruskal − Wallis

**Fig. 3:**
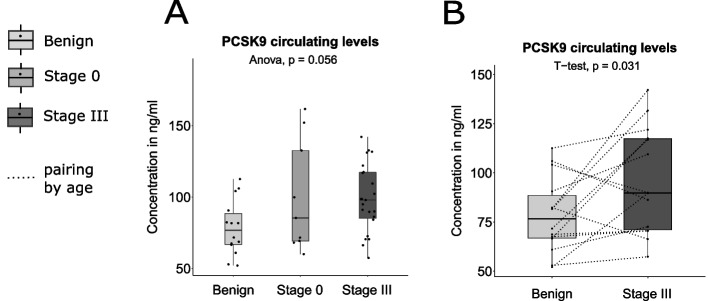
PCSK9 levels comparison between benign disease of the breast, stage 0 and stage III groups. **A** ANOVA analysis of PCSK9 levels between the three groups indicated an increase of PCSK9 with severity of the breast disease that did not reach statistical significance (*p* = 0.056). **B** Two-group comparison with Student T-test between benign disease of the breast and stage III breast tumor groups yielded a significant difference (*p* < 0.05) with higher PCSK9 levels in stage III breast cancer subgroup (95.9 ± 27.1 ng/mL, *n* = 14) compared to age-matched benign group (78.5 ± 19.3 ng/mL, *n* = 14). Dotted lines connect age-matched invididuals

Levels of Lp(a), ANGPTL3 and PCSK9 in the stage III breast cancer group and in the control group are presented in Table 2 and in Fig. 1. Concentrations of ANGPTL3 and Lp(a) were not different between stage III breast cancer and the control group (*p*-values of 0.33 and 0.51 for ANGPTL3 and Lp(a) respectively, Fig. 1A and B). However, PCSK9 levels were higher in the stage III breast cancer as compared to the control group, although the comparison did not reach statistical significance (*p*-value of 0.065, Fig. 1C). To verify if pooling cancerous (stage 0 breast cancers) with benign breast disease (non-cancerous lesions) to form the control group could have an impact on associations, we performed a stratification by pathology of the breast (benign disease and stage 0 tumor). No significant difference for ANGPTL3 and Lp(a) were obtained between groups (Fig. 2A and B). A near-significant increase in PCSK9 levels was obtained with breast disease severity (Fig. 3A , *p*= 0.056). A paired T-test performed between women with a benign pathology of the breast and age-matched stage III cancer subgroup indicated a significant increase in PCSK9 concentrations in the stage III cancer subgroup (95.9 ± 27.1 ng/mL) compared to women in the benign group (78.5 ± 19.3 ng/mL), as shown in Fig. 3B (*p*-value = 0.031, *n* = 14 in each group).

### Correlation between breast disease severity *versus* lipids, PCSK9, ANGPTL3 and Lp(a)

Next, we investigated the relationship between severity of breast disease and the lipid profile, PCSK9, ANGPTL3 and Lp(a) plasma levels, adjusted for age and BMI. No correlation was observed between the lipid profile (total cholesterol, triglycerides, HDL-C, LDL-C, non-HDL cholesterol or Apo B) and the severity of the breast disease (Table 3). The same observation was made for ANGPTL3 and Lp(a), for which no correlation was found with severity of the disease. A significant positive correlation appeared between PCSK9 levels and the severity of the breast disease (Rho Spearman = 0.34, *p* = 0.02, Table 3), a similar correlation as the one obtained before any adjustment (Rho Spearman = 0.34, *p* = 0.02).

**Table 3:** Severity of breast disease, lipid panel and PCSK9 levels: partial correlation analyses. Dependent variables were adjusted for age and BMI. No correlation between tumor stage (benign disease of the breast, stage 0 breast cancer, stage III breast cancer) and the lipid profile, ANGPTL3 or Lp(a) was obtained; however a significant correlation between PCSK9 levels and tumor stage, as measured by Spearman Rho coefficient, was obtained (*p < 0.05)

Controlled variables	Dependent variable	Spearman coefficient	*P*-value
**Age and BMI**	Cholesterol	-0.010	0.947
	Triglycerides	-0.033	0.833
	HDL-C	-0.147	0.342
	LDL-C	-0.004	0.980
	Non-HDL	-0.017	0.913
	Apolipoprotein B	-0.015	0.925
	ANGPTL3	-0.127	0.409
	Lp(a)	-0.043	0.781
	PCSK9	-0.339	0.024*

### Correlation analyses between all variables

**Fig. 4:**
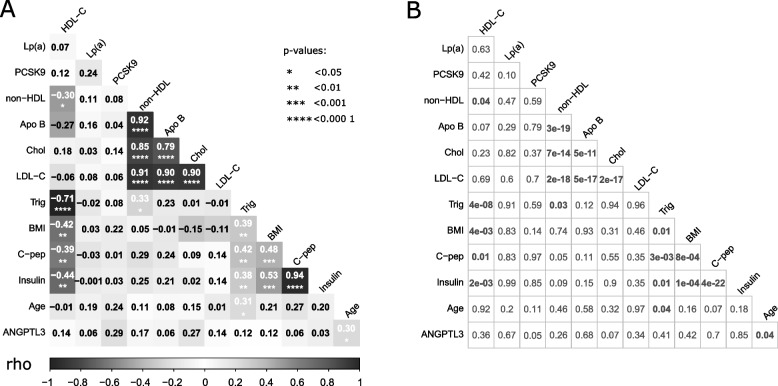
Correlation between all variables under study in the entire cohort. Relationship between age, body mass index (BMI), Insulin, C-peptide, triglycerides (Trig), Total cholesterol (Chol), HDL-cholesterol (HDL-C), non-HDL cholesterol (non-HDL), LDL-cholesterol (LDL-C), apolipoprotein B100 (Apo B), ANGPTL3, lipoprotein (a) (Lp(a)), and PCSK9 were assessed in the entire cohort. **A** Matrix on the left represents Spearman Rho correlation coefficients. Dark grey is indicative either of a Rho coefficient close to + 1 (positive correlation) or a Rho coefficient closer to -1 (negative correlation). White indicates an absence of correlation (coefficient equal to 0). **B** Matrix on the right displays the corresponding *p*-value for each Rho coefficient on the left. As expected, C-peptide, insulin and triglycerides show a significant correlation with BMI scores. Other statistically significant correlations include: LDL-C and its core protein Apo B, non-HDL cholesterol and LDL-C, non-HDL cholesterol and Apo B levels. Neither PCSK9, ANGPTL3 nor lipoprotein (a) show significant correlation with the rest of the lipid panel components. ANGPTL3 levels, however, appear to be significantly associated, at least partially (Rho = 0.30), with age (*p*-value = 0.04, *n* = 46)

We conducted a series of Spearman correlation analyses to investigate the existence of associations between the different parameters analyzed in our cohort (Fig. 4). Many well-documented associations were observed. Non-fasting insulin and its secretion by-product C-peptide showed a significant positive correlation with BMI (non-fasting insulin: Rho = 0.53, *p*-value = 0.0001; non-fasting C-peptide: Rho = 0.48, *p* = 0.0008) and with triglycerides (non-fasting insulin: Rho = 0.38, *p*-value = 0.009; non-fasting C-peptide: Rho = 0.42, *p*-value = 0.003). Similarly, triglycerides showed a positive correlation with BMI (Rho = 0.39, *p*-value = 0.008). HDL-cholesterol was negatively correlated to BMI (Rho = -0.42, *p*-value = 0.004) and to triglycerides (Rho = -0.71, *p*-value = 4 × 10^–8^). Non-HDL cholesterol, which accounts mainly for atherogenic particles, showed a strong significant correlation with LDL-C (Rho = 0.91, *p*-value = 2 × 10^–18^) and a correlation with triglycerides (Rho = 0.33, *p*-value = 0.02). Apolipoprotein B, which is the core protein of LDL and VLDL, showed a very strong correlation with non-HDL (Rho = 0.92, *p*-value = 3 × 10^–19^) and LDL-C (Rho = 0.90, *p*-value = 5 × 10^–17^).

As a less predictable outcome, a correlation between ANGPTL3 and age was found to be significant (Rho = 0.30, *p*-value = 0.04). No correlation was observed between the lipid panel and either ANGPTL3, Lp(a) or PCSK9.

## Discussion

In the present study, we investigated the levels of PCSK9, ANGPTL3 and Lp(a) in women with breast diseases of increasing severity (benign, stage 0 and stage III cancers). We also investigated whether these lipid-related proteins were associated to the lipid profile or breast disease severity. The main finding of this study is that PCSK9 levels tend to increase along with breast disease severity, a finding that warrants confirmation in a larger cohort.

### Severity of the breast disease *versus* the lipid panel

Although it is acknowledged that cholesterol is essential for tumor growth [61, 62], contradictory findings have been reported on the association between circulating lipoprotein levels and breast cancer risks [8, 11]. In the present cohort of women, none of the lipid parameters (total cholesterol, triglycerides, HDL-cholesterol, LDL-cholesterol, Apo B) was correlated to the severity of the breast disease (Table 3), a finding either explained by I) an insufficient power to detect changes in the lipid profile or II) a lack of association. Our study is in line with an analysis of the Women's Health Study that evaluated the prospective association of cancers (breast *n* = 864, colorectal *n* = 198, lung *n* = 190) with lipid profiles in 15,602 women. While lipids were associated with lung and colorectal cancer risks, no association was obtained between lipids and breast cancer risks [12]. Nevertheless, a study published in 2008 by Shah et al.including 125 women with breast cancer, 30 with benign lesions of the breast and 90 controls indicated that alterations in the lipid profile showed a significant correlation with breast cancer risks, disease status, and treatment outcome [63]. The results of Shah et al.were partly corroborated by a meta-analysis including fifteen prospective cohort studies involving 1,189,635 participants and 23,369 breast cancers. This meta-analysis suggested that triglycerides may be inversely associated with breast cancer risks while HDL-C might protect against breast cancer in postmenopausal women [64].

Clear evidence of a correlation between blood cholesterol levels and breast tumor histological features is still missing [65]. Co-existing physiological conditions, such as an underlying metabolic syndrome, post-menopausal status or chemotherapy, can influence the levels of circulating lipids and consequently blur the association between lipid profile and breast cancer prognosis [63, 66–68].

### ANGPTL3, Lp(a) and PCSK9 levels *versus* the lipid panel

No correlations were obtained between the levels of ANGPTL3, Lp(a) or PCSK9 and the lipid profile (Fig. 4A). The lack of association between Lp(a) levels and LDL particles is not an unexpected finding. Although apolipoprotein (a) is physically bound to LDL particles, Lp(a) concentrations are genetically determined [52] and are not correlated to LDL-C [69]. The lack of correlation between ANGPTL3 and the lipid profile (Fig. 4A) is a finding in line with a Finnish study that found ANGPTL3 serum protein concentrations did not predict lipid concentrations [70]. Similarly, despite an increase in PCSK9 levels between benign disease *vs* stage III cancers (Fig. 3B), no changes in LDL-C, non-HDL or Apo B were observed (Tables 2 and 3). This observation was consistent with correlation studies between baseline PCSK9 levels and cholesterol measurements in 55 patients with different stages of lung cancer [71]. While PCSK9 levels increased with cancer stage, cholesterol levels did not follow the same trend. We propose that either the rise of PCSK9 levels in our study was too small to lead to a significant increase in LDL-C levels, or that it did lead to a transient rise in LDL-C levels that was compensated by the uptake of LDL-C by cancer cells. Our study is not designed to explore these processes.

### Breast disease severity *versus* ANGPTL3, Lp(a) and PCSK9 levels

Severity of breast cancer is assessed using a staging system based on how large the primary tumor is and how far it has spread within the body. Stages 0 to II tend to have a better long-term outcome compared to stages III to IV, which are qualified as “high-stage” cancers.

In our study, no statistical differences in ANGPTL3 or Lp(a) levels were observed between groups of increasing breast disease severity (Table 3, Figs. 1 and 2). This lack of association could be attributed to a low statistical power or a real absence of correlation with disease severity. The observation of a significant association between severity of breast disease and PCSK9 levels (Table 3, Fig. 3B) points toward a potentially important effect of PCSK9 in cancer, the mechanism of which deserves to be further investigated.

Our findings are in line with results reported in other human cancers. A recent in vivo study has shown that patients with gastric cancers had higher plasma levels of PCSK9 compared to age-matched healthy controls [72]. Other studies have advocated that PCSK9 could be a prognostic marker for advanced non-small cell lung cancer [73] and for response to immune checkpoint inhibitors therapy [71]. Combined with the present results, these studies support the need to confirm the association, if any, between PCSK9 elevation and enhanced cancer aggressivity.

### Origin of circulating PCSK9

Our study was not designed to address the origin of the extra levels of PCSK9 measured in advanced breast cancers. A major organ responsible for PCSK9 levels in circulation is the liver, which is in charge of regulating cholesterolemia. A possibility would be that tumoral cholesterol uptake leads to a transient decrease in cholesterolemia which could trigger hepatic secretion of PCSK9. Higher PCSK9 levels would compensate tumoral cholesterol uptake and maintain cholesterolemia, without any apparent changes in the lipid profile, as observed in our study. Whether or not circulating levels of PCSK9 can partly originate from the tumor itself is an open question that needs to be addressed.

### PCSK9 and tumor progression

A recent study by Suh et al.showed that nucleoprotein Ahnak mediated B16F10 melanoma cells metastasis in mice via enhanced PCSK9 expression [74]. Actual data tend to support a pathogenic and pro-tumoral role for PCSK9, by both maintaining cholesterol supplies and by promoting MHC-I degradation on tumor cells, favoring immune escape of tumors [34]. Whether the increase in PCSK9 plays an active role in the progression of breast cancer requires further investigation. Comparing tumoral levels of PCSK9, MHC-I and HLA-C using standard methods (ex: ELISA) in tumors of increasing disease severity could help decipher the role of PCSK9 in tumors.

### Limitations

#### Availability of samples, size of the cohort, Type I and II errors

The present study was limited by the availability of plasma samples (fourteen at the benign stage, nine at the premalignant stage and twenty-three stage III breast cancers), therefore conclusions could be biased by the small sample size. The increase in PCSK9 with breast disease severity could be observed by chance rather than reflect a real difference in PCSK9 levels (Type 1 error, rejecting the null hypothesis). Nevertheless, it has been reported that significant results in small cohorts with narrow confidence intervals have a good predictable value for reproducibility in larger groups [75], making associations in small cohorts valuable to report. The sample size could also be insufficient to reveal associations between ANGPTL3, or Lipoprotein (a), and the severity of the breast disease. Small sample size increases the probability of dismissing true differences between groups deemed non-significant by *p*-values > 0.05 (Type 2 error, failing to reject the null hypothesis). In our small cohort of women, the presence of an association between breast disease severity and PCSK9 levels, and the lack of association between breast disease severity and ANGPTL3 or Lp(a) levels, warrants further investigation of these lipid-related proteins in larger cohorts of women with and without breast cancers. Of note, we performed these correlation studies whilst controlling for age and BMI as possible confounders.

#### Combination of benign disease with premalignant tumours to form a control group

Due to the limited availability of samples, the control group combined women without cancers (benign pathology of the breast) with samples from women with a stage 0 breast cancer. While such a merge of non-cancerous samples with early-stage cancerous samples to form a control group may have blurred associations between PCSK9 and breast disease severity (Fig. 1C), a statistically significant association was obtained once samples were stratified according to breast disease severity (Fig. 3B and Table 3).

#### HDL cholesterol

A statistically significant difference in HDL-cholesterol (1.3 mmol/L vs 1.8 mmol/L, *p* = 0.01) was observed between the benign disease of the breast group and the stage 0 breast cancers, although interquartile ranges (accompanying median values) and standard deviation (associated to mean values) overlapped (Table 1). This result can be due to chance since the sample sizes are small (*n* = 14 for benign disease, and *n* = 9 for stage 0 breast cancer). HDL is not associated with PCSK9 (Fig. 4A) and therefore this difference is not likely to invalidate our findings. HDL values in all groups are in the normal range for women (1.3 mmol/L and above).

#### Non fasting lipids, LDL-cholesterol and effect on correlations

LDL-C levels were calculated by applying the Friedewald equation, which is aimed to be used in people with normal triglycerides values (below 1,8 mmol/L). Available blood samples were from non-fasting subjects, nevertheless mean triglycerides were at 1,8 mmol/L or below (Table 1 and 2). For individual participants having triglycerides above 1,8 mmol/L, circulating LDL-C may have been underestimated. As a mitigating strategy, Apo B and non-HDL levels were obtained (Table 1, Fig. 4); both measurements are unaffected by the fasting/non-fasting status of participants. Of interest, the 3 analytes reflecting beta-lipoproteins gave similar rho values and non-statistically significant correlations with PCSK9 (LDL: rho = 0.06; *p* = 0.70; non-HDL: rho = 0.08; *p* = 0.59; Apo B: rho = 0.04; *p* = 0.79). Of note, Apo B is measured using antibodies (immunoturbidimetry) while cholesterol (Total and HDL) is measured by a combination of enzymatic and chemical methods (non-HDL = Total cholesterol – HDL cholesterol). The fact that analytes reflecting beta-lipoproteins and measured by different techniques give similar correlations permits cross-validation of our results.

#### Potential confounding variables affecting PCSK9 levels

Statins and estrogens are known factors that modify PCSK9 levels. Information on medication prescribed to participants, including statins, other lipid-lowering drugs or hormonal treatments were not available from the biobank. While menopausal status of participants were available for the stage 0 and stage III breast cancers, the information was lacking for participants in the “benign disease of the breast” group. The absence of information on statins, other lipid-lowering drugs, hormonal replacement and the menopausal status, all variables which affect PCSK9 levels and circulating lipid levels, is a limitation of our study. Finally, ethnical origins of women were not documented, and it is still unclear if PCSK9 levels are similar in people from different ethnicities.

## Conclusions

In summary, in this small cohort of 46 women, PCSK9 levels tended to increase with the severity of the breast disease. This association was not observed for Lp(a) and ANGPTL3, two other lipid-related circulating proteins. Considering the key role of PCSK9 on lipid metabolism (hyperlipidemic factor) and immune response (destruction of MHC-I), confirmation of increased PCSK9 levels with breast cancer severity in a larger cohort of patients is indicated.

## Data Availability

The datasets analyzed for the current study are not publicly available (confidentiality issues) but can be made available by the corresponding author upon reasonable request.
