# Correction: Circulating levels of PCSK9, ANGPTL3 and lp(a) in stage III breast cancers

**DOI:** 10.1186/s12885-024-13182-w

**Published:** 2024-11-15

**Authors:** Emilie Wong Chong, France-Hélène Joncas, Nabil G. Seidah, Frédéric Calon, Caroline Diorio, Anne Ganglof

**Affiliations:** 1 Faculty of Medicine, Laval University, Quebec City, QC Canada; 2 Oncology Research Axis, CHU de Québec-Laval University Research Center, Quebec City, QC Canada; 3 Cancer Research Centre (CRC), Laval University, Quebec City, QC Canada; 4 Laboratory of Biochemical Neuroendocrinology, Institut de Recherches Cliniques de Montréal, Montreal, QC Canada; 5 Faculty of Pharmacy, Laval University, Quebec City, QC Canada; 6 Neuroscience Research Axis, CHU de Québec-Laval University Research Center, Quebec City, QC Canada; 7 Centre Des Maladies du Sein, Hôpital du Saint-Sacrement, Quebec City, QC Canada; 8 Lipid Clinic, CHU de Québec, Quebec City, QC Canada

**Correction: BMC Cancer 22, 1049 (2022)**

10.1186/s12885-022-10120-6

Following publication of the original article [1], the authors reported an error in the Spearman’s correlation coefficients table in which all coefficients values were incorrectly set to negative.

Incorrect Table 3:

**Table:** 

Controlled variables	Dependent variable	Spearman coefficient	*p*-value
Age and BMI	Cholesterol Triglycerides HDL-C LDL-C Non-HDL Apolipoprotein B ANGPTL3 Lp(a) PCSK9	− 0.010 − 0.033 − 0.147 − 0.004 − 0.017 − 0.015 − 0.127 − 0.043 − 0.339	0.947 0.833 0.342 0.980 0.913 0.925 0.409 0.781 0.024*

Correct Table 3: Only cholesterol, non- HDL and apolipoprotein B were negatively correlated with independent variable “tumor stage” in the study.

**Table:** 

Controlled variables	Dependent variable	Spearman coefficient	*p*-value
Age and BMI	Cholesterol Triglycerides HDL-C LDL-C Non-HDL Apolipoprotein B ANGPTL3 Lp(a) PCSK9	− 0.010 0.033 0.147 0.004 − 0.017 − 0.015 0.127 0.043 0.339	0.947 0.833 0.342 0.980 0.913 0.925 0.409 0.781 0.024*
